# Adaptation of a brief smoke-free homes intervention for American Indian and Alaska Native families

**DOI:** 10.1186/s12889-019-7301-4

**Published:** 2019-07-23

**Authors:** Katherine M. Anderson, Michelle C. Kegler, Lucja T. Bundy, Patricia Henderson, June Halfacre, Cam Escoffery

**Affiliations:** 1 Department of Behavioral Sciences and Health Education, Emory Prevention Research Center, Rollins School of Public Health, Emory University, Atlanta, GA USA; 2 Black Hills Center for American Indian Health, Rapid City, SD USA; 3 Cherokee Nation, Tahlequah, OK USA

**Keywords:** American Indian, Alaska native, Tobacco, Smoke-free homes, Secondhand smoke exposure

## Abstract

**Background:**

The goal of adaptation is to maintain the effectiveness of the original intervention by preserving the core elements that account for its success while delivering an intervention that is tailored to the new community and/or cultural context.

The current study describes the process of adapting an evidence-based smoke-free homes (SFH) intervention for use in American Indian/Alaska Native (AI/AN) households.

**Methods:**

We followed a systematic adaptation process. We first assessed the community through focus groups coordinated in collaboration with tribal partners. Because our team included the original developers of the intervention, the steps of understanding the intervention, selecting the intervention and consulting with experts were simplified. Additional steps included consulting with stakeholders through a national work group and collaboratively deciding what needed adaptation.

**Results:**

A number of key themes pertinent to the adaptation of the SFH intervention were identified in the focus groups. These included the gravity of messaging about commercial tobacco use; respect, familialism, and intergenerationalism; imagery, including significant symbolism, colors, and representative role models; whether and how to address traditional tobacco; and, barriers to a SFH not adequately addressed in the original materials.

**Conclusions:**

Adaptation of an intervention to create smoke-free homes in AI/AN families necessitated both *surface structure* changes such as appearance of role models and *deep structure* changes that addressed core values, and beliefs and traditions.

**Electronic supplementary material:**

The online version of this article (10.1186/s12889-019-7301-4) contains supplementary material, which is available to authorized users.

## Contributions to literature

- This study illustrates a participatory process for adapting a research-tested intervention with tribal partners. High quality adaptation processes conducted collaboratively with tribal partners are relatively rare.
- The process aligns with common adaptation steps identified in a recent scoping review on adaptation of evidence-based interventions.
- Given traditional uses of tobacco in American Indian communities, both surface and deep structure changes were required in this cultural adaptation of a research-tested intervention.

## Background

The need to adapt evidence-based interventions to local context and culture is well-established [1]. Cultural adaptation starts with interventions known to be effective in a different setting and/or population and systematically modifies them to “consider language, culture, and context in such a way that is compatible with the client’s cultural patterns, meaning, and values” (p. 362) [2]. The goal of adaptation is to maintain the effectiveness of the original intervention by preserving the core elements that account for the intervention’s success while delivering an intervention that is tailored to the new community and/or cultural context. Changes are grouped into two categories: *surface structure* changes, or changes such as “ethnicity or appearance of role models” in the intervention, and *deep structure* changes, or “changes addressing the core values, beliefs, norms, and other more significant aspects of the cultural group’s world views and lifestyles” [3, 4].

Particularly, when adaptation consists of changes to surface structure and not content, pedagogy, and implementation, the validity of the intervention is unlikely to be compromised, allowing for an intervention that is more culturally appropriate and which incorporates known cultural values and experiences while maintaining effectiveness [3, 5–7]. Such culturally adapted interventions often have a greater effect than the original intervention, with adaptations specific to single group rather than to a general population [8]. For American Indian/Alaska Native (AI/AN) communities, culturally tailored interventions have been found to be more memorable than and are preferable to interventions targeting the general population [9–11].

Quite a bit has been written about the need to adapt tobacco control interventions for AI/AN communities. For many tribes, traditional tobacco plays an important role in spiritual and cultural traditions, and is part of ceremonial, medicinal and religious practices [12, 13]. Distinguishing between recreational use of commercial tobacco and sacred use of tobacco is essential in culturally appropriate tobacco control efforts [12, 13]. Moreover, language commonly used in tobacco control, such as tobacco-free, can be seen as culturally insensitive in a tribal context [13].

The Smoke-Free Homes: Some Things are Better Outside intervention is a brief intervention developed to promote smoke-free home policies among low-income households [14]. Smoke-free homes are associated with decreased exposure to secondhand smoke (SHS), and increasingly, smoking cessation [14–17], thus contributing to both health and economic benefits [18]. The Smoke-Free Homes: Some Things are Better Outside intervention has been shown to successfully facilitate the establishment of home smoking rules in a series of five studies: a pilot test, an efficacy trial, an effectiveness trial, a generalizability trial, and a dissemination study, in collaboration with 2–1-1 call centers in Georgia, North Carolina, Texas, Oklahoma, Ohio, Alabama and Florida [15, 19, 20]. Participants in these studies were predominantly African American, White, and Hispanic.

The program was developed for a general population and did not address the complexities of tobacco use in Native communities stemming from traditional ceremonial uses and historical context. Although national data on smoke-free homes among AI/AN families are not available, a higher smoking rate than the general U.S. population suggests that smoke-free homes may be less common given smokers are less likely to have a smoke-free home than nonsmokers [21]. In 2016, 31.8% of AI/AN adults smoked, compared to 15.5% of the general adult population in the U.S. [22]. Notably, AI/AN women smoke at higher rates than AI/AN men, and they are the sub-population with the smallest decrease in prevalence from 2005 to 2015 [23].

The aim of this current study is to adapt the evidence-based intervention “Smoke-Free Homes: Some Things are Better Outside” for use in AI/AN households, to ultimately increase the prevalence of smoke-free homes and reduce SHS exposure among AI/AN children and adult nonsmokers. This paper describes the adaptation process and the resulting modifications made to the intervention and may provide a blueprint and considerations for cultural adaptation of other tobacco control interventions, both within the context of the United States and globally among indigenous cultures utilizing sacred or traditional tobacco.

## Methods

### Description of the original intervention

The Smoke-Free Homes: Some Things are Better Outside intervention consists of four components (core elements), including three mailings of print materials and one coaching call, targeting both smokers and nonsmokers living in a household without a smoking ban. The intervention is theory-based [24] and utilizes persuasion, role modeling, goal setting, environmental cues, and reinforcement to help participants establish and maintain a smoke-free home through five steps: 1) deciding to create a smoke-free homes, 2) talking to household members about making a home smoke-free, 3) setting a date for going smoke-free, 4) actually making a home smoke-free, and 5) keeping the home smoke free. The materials are visually appealing and the intervention uses humor to illustrate that cigarettes, along with other behaviors (e.g., washing a dog, garbage bags) belong outside, with a cute dog and young African American boy providing consistency across mailings and materials. Visuals and messages were designed to appeal to the general population, and were developed with input from partners and community members in Georgia, who were primarily African American and White.

### Overview of the adaptation process

We used a multi-step process for the Smoke-Free Homes program adaptation which aligns with a framework proposed by Escoffery et al., derived from a scoping study of frameworks for adapting evidence-based interventions (EBIs) [25]. Steps in the framework include: 1) assess the community, 2) understand the EBI, 3) select the EBI, 4) consult with experts, 5) consult with stakeholders, 6) decide on needed adaptations, 7) adapt the original EBI, 8) train staff, 9) test the adapted materials, 10) implement the adapted EBI, 11) evaluate. For this adaptation process, steps 1–7 have been completed, with the remaining steps forthcoming. Descriptions of each completed step, as well as how the step was conducted in the adaptation process, are presented in Table 1. Briefly, we assessed the community through focus groups coordinated in collaboration with tribal partners. Because our team included the original developers of the EBI, steps 2 (understanding the intervention), 3 (selecting the intervention), and 4 (consult with experts) were simplified. Step 5 is to consult with stakeholders and Step 6 is to decide what needs adaptation.

**Table 1:** Adaptation Process based on Adaptation Guidance

	Step Name	Step Description	Step Activities
Formative Research Phase	1. Assess Community	• Identify behavioral determinants and risk factors of the new target population using focus groups, interviews, needs assessments, and logic models • Assess organizational capacity to implement the program	Conducted ten focus groups with five tribal commununities- one smoking and one non-smoking each. Topics included barriers to EBI application and success, cultural differences impacting EBI, surface and deep changes to materials necessary
	2. Understand the Intervention	• Identify and review relevant EBIs and their program materials • Understand the theory behind the programs and their core elements	The majority of EBIs focused on smoking rules target multi-unit housing or public places, not single family homes; to our knowledge, no EBI focusing on smoke-free homes has been adapted to AI-AN populations. Most AI-AN adaptations seek to change smoking behavior itself, rather than smoke-free home rules. Additionally, adaptation team included original developers of the intervention at the Emory Prevention Research Center (EPRC)
	3. Select the intervention	• Select the program that best matches the new population and context	SFH is an EBI with success in multiple contexts, and one of few interventions that seeks to change smoking rules in the home environment to reduce exposure to SHS and THS, and may be a pathway to quitting. Additional support for the intervention was given by focus group participants, regarding perceived acceptability and efficacy.
Adaptation Phase	4. Consult with experts	• Consult with content experts, including original program developers, as needed • Incorporate expert advice into program	EPRC is home to experts on tobacco control research, smoke-free homes, and adaptation of EBIs. Additionally, it is both the source of the original intervention and this adaptation. Therefore, the members of the EPRC act as experts for this adaptation. Additionally, focus group participants are expects on their communities, as members, and as such were consulted regarding changes.
	5. Consult with Stakeholders	• Seek input from advisory boards and community planning groups where program implementation will take place • Identify stakeholder partners who can champion program adoption in new settings and ensure program fidelity	Establishment of AI-AN workgroup consisting of 16 members, including tribal partners from Michigan, Oklahoma, California, and Alaska. Tribal partners served to give input on adaptation materials and support future dissemination activities.
	6. Decide what needs adaptation	• Decide whether to adapt or implement the original program • Determine how original and new target population/setting differ in terms of risk and protective factors • Identify areas where EPI needs to be adapted and include possible changes in program structure, content, provider or delivery method	Based on focus group results, previous literature, and consultation with the work group, decision was made to make surface-level changes to the materials, including most notably the adaptation of visuals and imagery to include primarily AI-AN individuals from diverse cultures, as well as images of nature, and AI-AN associated symbols, such as the medicine wheel. Additionally, some changes to language were made to emphasize family and community, as well as respect. Program structure, content, and delivery remained the same to ensure validity.
	7. Adapt the original program	• Develop adaptation plan • Adapt the original program contents through collaborative efforts • Make culture adaptations continuously through pilot testing • Core components responsible for changes should not be modified	Materials were adapted in collaboration with an American Indian designer able to make changes that were culturally appropriate. Changes were reviewed by the adaptation team as well as the adaptation AI-AN work group, and revised as appropriate.

Using a community-engaged approach, we consulted with stakeholders as part of Step 1. We began the adaptation process by establishing a work group. The work group comprised tribal partners from across the U.S., including those based in Michigan, South Dakota, Oklahoma, California, and Alaska. Members of the work group included individuals we knew through existing collaborations and those responding to an invitation sent to members of the Centers for Disease Control and Prevention’s Native Tobacco Network (https://keepitsacred.itcmi.org/). The work group advised on all stages of the process, including the decision to conduct focus groups, development of the focus group discussion guides, development of draft materials for focus group review, logistics and recruitment of community members for the focus groups, review of preliminary results, and revision of print materials [26].

### Description of the focus groups

#### Recruitment of focus group participants

Work group members in California, Oklahoma, Michigan and Alaska volunteered to host focus groups in their communities. They coordinated focus group logistics, including location and time, and recruited focus group participants. Primary recruitment methods included flyers, social media outreach, and word-of-mouth outreach. Eligible participants were 18 years of age or older and self-identified as American Indian or Alaska Native. There were 10 focus groups total, with each community hosting between two and four focus groups, with smokers and nonsmokers in separate groups. Between 5 and 15 participants attended each focus group, resulting in 95 total participants. Focus groups were held in tribal or partner organization facilities. Focus groups lasted about 90 min, and compensation was provided to participants in the form of a $40 gift card. See Additional file 1 for the Standards for Reporting Qualitative Research checklist [27].

#### Focus group guide and participant survey

The focus group guide was developed in collaboration with members of the work group and covered establishment and maintenance of smoke-free home rules, facilitators and barriers to a smoke-free home, exceptions to a ban, thirdhand smoke, electronic cigarettes, marijuana, and commercial tobacco use at home. Additionally, we discussed cultural elements that may impact smoke-free home rules, including respect for elders and traditional use of tobacco, and we asked focus group participants to review the original Smoke-Free Homes print materials and crude mock-ups of some alternatives (e.g., child playing basketball indoors) with the same theme of “Some Things are Better Outside” and a new theme recommended by the work group “Respect our Past, Protect our Future.” Our general findings related to establishing and maintaining a smoke-free home are reported elsewhere [26].

#### Analysis

Focus groups were audio-recorded, transcribed and then checked for accuracy against the audio recording. Two members of our team then reviewed the transcripts to identify inductive themes that emerged from the data and deductive themes based on the focus group guide. These preliminary analyses informed a codebook which was used for independent coding of each transcript. Analysts then compared codes and resolved any discrepancies through discussion. Finalized codes were entered into NVivo qualitative analysis software, then themes were assessed for saliency across focus groups stratified by smoking status [28]. We identified sub-themes, organized them within the main themes, and extracted representative quotes. Preliminary results were presented to the work group for interpretation and to make decisions about adapting the materials.

## Results

### Key themes with implications for adapting the intervention

A number of key themes pertinent to the adaptation of the SFH intervention were identified. These include the gravity of tobacco messaging; respect, familialism, and intergenerationalism; imagery, including significant symbolism, colors, and representative role models; whether and how to address traditional tobacco; and barriers to a smoke-free home not adequately addressed in the original materials.

#### Gravity of tobacco messaging

Focus group participants felt that messaging surrounding SFHs, commercial tobacco use, and traditional tobacco should not include humor, because of the gravity of the subject itself. The original Smoke-Free Homes materials include a comedic theme including the placement of items, pets, and children in the home who are doing things that disrupt the indoor environment, with the theme “Some Things are Better Outside.” AI/AN participants generally reacted negatively to this, saying, for example, “*I feel like if you’re talking about using [tobacco] as traditional, it shouldn’t be funny anyways. It should be more serious*” (Michigan Nonsmoker (NS). This sentiment was repeated several times. Others found that the theme did not resonate, for example, saying, “*I don’t think it’s funny. I don’t think a dog peeing on the couch is funny. It wasn’t funny to me*” (California Smoker).

#### Respect, familialism and intergenerationalism

Respect was a cross-cutting theme in discussions regarding tobacco, particularly as a reason participants did not allow smoking in their home, a reason they were hesitant to smoke in the homes of others, or as a justification for *not* asking particular individuals, including elders, to stop smoking in their home. Participants identified respect as a powerful theme for messaging, particularly when combined with the importance of familialism and intergenerationalism. These manifested in sharing and passing on knowledge, respecting elders and the history of communities, and ensuring the health of future generations. As one participant said, “*We have stories that we’ve told through generations, and … there’s always a really important message*” (California NS). Referring to the use of respect, familialism, and intergenerationalism in messaging about the “Respect the Past, Protect our Future” mock-up, another participant said,

*“I like it because it’s respecting like -- it has like elders up here and it’s saying respect our past, and then of course everybody -- the reason why we all don’t allow smoking in our homes is for the benefit of our children’s health, I think everybody would agree that we want to protect our children, protect our children’s health, and that’s -- I think everybody would agree that we want to protect our children, protect our future, protect our health. ... Because without the children, there’s no future of the tribe.” (Michigan NS).*

#### Imagery

##### Significant symbolism and colors

Focus group participants noted in their review of the original and mocked-up SFH intervention materials that they would prefer the use of recognizable imagery, symbolism, and colors, so as to customize the materials better for their communities. This included images of nature- “*Put the trees, put the river out there but -- mountain, something outside* ” (California Smoker)- and particularly nature that was recognizable to each community receiving the materials: “[You can use] *the general* [respect theme mailing] *that was in the envelopes and then for each region, for each like population that you’re targeting, you can just add a little* [regional] *thing*” (California NS).

Additionally, focus group participants recommended the use of the medicine wheel imagery within the materials, both to customize them to AI/AN communities, and to bring forward the emphasis on health. Similarly, use of the colors of the medicine wheel- red, yellow, black, and white- were suggested for integration into the color scheme of the materials.

##### Representativeness

The importance of role models and images of people within the SFH materials that are identifiably AI/AN was emphasized throughout the focus groups, not only for customizing the materials to be applicable to their communities, but also to show children within their community role models they can relate to. One participant expressed the importance of this both to her and her son, saying:

*“…it’d be nice to have things individually tailored to each area, but if I opened a thing and was just like oh my gosh, this is made for Natives, I mean, I’d just be so blown away, you know, because now my son watches TV and he’ll say there’s an Indian on -- I’ll be like well, that’s really a Mexican, but I’m glad that you’re excited … So I think yeah, it’d be good to have an individual component, but at the same time if you just got something that was like wow, they took the time to make this for a Native, you know, I think that that would kind of be like -- because we’re usually just -- not a race, but – [other].” (California NS).*

A related theme was the recommendation to acknowledge diversity within tribal communities and that some AI/AN families have members with a range of racial and ethnic backgrounds.

#### Traditional tobacco

Participant’s opinions varied as to whether or not traditional use of tobacco was pertinent to an intervention to create SFHs; however, most came to the conclusion that while they would not consider smoke created from the use of traditional tobacco to fall under SFH rules, it should still be addressed within the intervention materials. One participant commented, *“There are a lot of campaigns already about keep tobacco sacred, so I think if you add* [something about traditional tobacco], *I think that would be a start for sure”* (California NS). Similar comments were heard in other focus groups: “*I would say material like this is -- would be pretty accepted in tribal communities. … saying use tobacco in a sacred way or … things like that, like you’ll see it from time to time, but for like on a national level, something like this would be effective, I’d say*” (Michigan Smoker).

#### Cold weather as a barrier

Finally, participants in the focus groups brought up a previously unaddressed barrier to SFH creation- the difficulty of smoking outside in cold weather (as opposed to bad weather in the original materials). A participant described this, saying it reduced their tobacco intake: “*Really, like if it’s raining or if it’s cold, I don’t go outside. I don’t smoke. So typically in the winter I’m not a smoker unless I’m at the casino. So yeah, so if I smoke at home, I’m on my porch or my sister’s porch, yeah*” (Oklahoma Smoker). Alternatively, another participant described how this prevented them from establishing a SFH home, saying,

*“Well, if we’re sitting there in the middle of winter and we have people over, I’m like listen, if you want to go away from us from smoking, because they will, that is the norm. You don’t go outside. Just go in the living room or in the kitchen, and then my house is very large, and by now we just already know the problem.” (Michigan NS).*

### Adaptations made to the intervention materials

Following analysis of the focus group data and consultation with the work group, we hired an American Indian graphic designer to make an adapted set of intervention materials. The changes included both surface changes and deep changes, as outlined in Table 2. Overall, messaging was changed to remove humor; a new theme of “Respect Our Past, Protect Our Future” was added; respect in general and particularly in relation to intergenerationalism and familialism was emphasized; imagery throughout the materials was altered to reflect culturally applicable people, symbolism, and color schemes (e.g., nature, medicine wheel); strategies for dealing with cold weather were added; and, an additional insert on the use of traditional tobacco was developed for use as appropriate. Table 3 outlines the specific materials included in the adapted intervention, as well as which alterations described in Table 2 are applicable for each intervention material.

**Table 2:** Overview of Changes to the Intervention

Surface Changes	Deep Structure Changes
1. No use of humor in messaging	1. Theme change from “Some things are better outside” to “Respect Our Past, Protect Our Future”
2. Images of AI/AN people	2. Language change to emphasize familialism and intergenerationalism
3. Imagery of nature, scenery familiar to specific tribes	3. Respect-focused language
4. Use medicine wheel imagery and colors	4. Creation of traditional tobacco insert
5. Addition of cold weather barrier

**Table 3:** Adapted Intervention Materials

	Material	Description	Applicable Changes
General Materials	Covers (*n* = 2)	Folders that double as envelopes for mailing 1. Depicting images of AI/AN individuals and families, with themes of nature and “Respect our Past, Protect Our Future,” respectively.	Theme change from “Some things are better outside” to “Respect our Past, Protect Our Future”; No use of humor in messaging; Images of AI/AN people, Language change to emphasize familialism and intergenerationalism; Imagery of nature, scenery familiar to specific tribes; Use medicine wheel imagery and colors; Respect-focused language.
	Envelopes (*n* = 5)	Envelopes used for mailings 2 and 3, which depict different nature scenes, including the plains, mountains, great lakes, pine forests, and deciduous forests, respectively.	Imagery of nature, scenery familiar to specific tribes. Removal of humor messaging.
Mailing 1	5-Step Guide	A guide that outlines the five steps to creating a smoke-free home.	No use of humor in messaging; Removal of dog images; Images of AI/AN people; Language change to emphasize familialism and intergenerationalism; Use medicine wheel imagery and colors.
	Stickers		No use of humor in messaging; Use medicine wheel imagery and colors.
	Window Cling	A window cling that states “This is a smoke-free home”	Use medicine wheel imagery and colors.
	Traditional Tobacco Insert	An insert that addresses the difference between commercial tobacco and traditional tobacco.	Use medicine wheel imagery and colors; Creation of Traditional Tobacco insert.
Mailing 2	Challenges & Solutions Factsheet	A factsheet that addresses multiple challenges individuals seeking to make their home smoke-free might face, as well as solutions to overcome those challenges.	Images of AI/AN people; Use medicine wheel imagery and colors; Addition of solutions for cold weather as a barrier.
	Photonovella	Comic-style story of a family who works to make their home smoke free to improve a child’s health.	Images of AI/AN people; inclusion of a father figure.
	E-Cigarette Insert	An insert that addresses the health impacts of using e-cigarettes.	Use medicine wheel imagery and colors.
Mailing 3	Smoke-Free Homes Newsletter	A newsletter containing stories of individuals, couples, and families who made their homes smoke-free, as well as why and how.	Images of AI/AN people; Language change to emphasize familialism and intergenerationalism; Respect-focused language.
	Stickers		No use of humor in messaging; Use medicine wheel imagery and colors.
	Window Cling	A window cling that states “This is a smoke-free home.”	Use medicine wheel imagery and colors.
	Thirdhand Smoke Insert	An inset that addresses the health impacts of thirdhand smoke.	No changes.
	Cigar Insert	An insert that addresses the health impacts of using cigar, little cigars, or cigarillos.	No changes.

## Discussion

This paper describes the process of cultural adaptation of an EBI focused on creating smoke-free homes. Alterations made to the SFH intervention are derived from the recommendations made and themes discussed by focus group participants in five diverse AI/AN communities, as well as with consideration for the previous body of literature on cultural adaptations for AI/AN populations. Many of these changes revolved around content, persons, and context as specified in cultural adaptation models [2]. These changes were made to increase the acceptability of the intervention among AI/AN populations, as well as to better meet the needs of AI/AN communities. Such changes are in line with recent thinking regarding cultural adaptations [29], suggesting that tobacco-related interventions with AI/AN populations should make an effort ensure that messaging is culturally relevant, and that interventions may be perceived as more applicable to the priority population if the messaging incorporated evident understanding of the social and cultural factors involved in smoking behavior. Similarly, Gould et al. determined that, based on their systematic review, resources for campaigns against commercial tobacco use should include “appropriately diverse cultural elements,” including depictions of customs and perspectives from a range of tribal nations [10].

The changes made to the SFH materials span the scope of the intervention. Select changes were made in response to direct commentary by the focus groups regarding the original intervention materials, such as the alteration in messaging to exclude humor, the use of language focusing on respect, and the integration of nature imagery. Other changes, however, reflect broader recommendations for appropriate cultural adaptation of interventions for AI/AN communities.

The importance of visual depictions of AI/AN individuals and communities, acknowledging diversity within and across these communities, and accuracy and contemporaneity of these depictions has been noted repeatedly in literature on interventions in AI/AN populations, and is confirmed once again in this adaptation [10, 30–33]. Familialism was discussed and emphasized in the current research, taking multiple forms supported by previous literature: the influence of elders and intergenerational learning [11], the importance of depictions of family [31, 34], and the utility of targeting familialism and intergenerationalism in smoking interventions [31]. A preference for cultural design elements that are familiar to AI/AN populations, including colors reflective of AI/AN traditions has also been referenced in literature, and was re-iterated in our findings [10, 33].

While there is clear support in the literature for addressing sacred uses of tobacco in intervention materials targeted towards AI/AN communities, [29–31, 35] the focus groups in our formative research found this to be contentious, with mixed opinions on whether or not it was appropriate to include in educational materials. While the ultimate consensus was that traditional uses of tobacco should not be neglected, concerns were iterated that traditional tobacco should not be associated with concerns about SHS in the home or commercial smoking, or did not need to be addressed given sufficient knowledge in communities. Despite the decision to acknowledge traditional uses of tobacco in the adapted SFH materials, inclusion of traditional uses of tobacco in future interventions may require further thought and consultation with tribal partners.

While many of these themes are relevant across tribes, we acknowledge the heterogeneity of culture across tribes, including traditional uses of tobacco, prevalence of smoking, language, beliefs, and history. Because it would be cost-prohibitive to create a completely different set of materials for each tribe interested in using the intervention, we have developed a general AI/AN set of materials with pieces that can be tailored to local tribal culture, including the envelopes that depict natural scenes from different regions of the country (e.g., mountains, plains, lakes) and an insert that can be modified to accurately portray traditional uses. The window cling can also be easily adapted for specific tribes, allowing for modifications for personal context or environment [2]. Interestingly, the Alaska Native focus group participants suggested that it may be more appropriate for a general Alaska version of the materials than to group them with American Indians in the lower 48 states, in part due to not using tobacco traditionally.

Our approach to adaptation has a few limitations. All of our focus groups were conducted in rural areas, as a result, we did not obtain perspectives from urban AI/AN. This is an important omission given that a significant proportion of the AI/AN population lives in urban areas. A second limitation stemmed from the quality of the materials we presented in the focus groups. While they were sufficient for generating a reaction, after the first couple of focus groups we knew that the reaction would generally be negative. For the “Some Things are Better Outside” theme, it was difficult to know if participants were reacting to the theme itself or the low-quality draft materials. As in all focus groups, the dynamic may have also influenced the group, with more outspoken member leading the group toward or away from a particular reaction. In one community, the focus groups were very large making it difficult to manage the group dynamics and ensure that all voices were heard. Lastly, the moderator was an outsider as a non-Native faculty member from Atlanta. While local partners were present, the outsider status of the moderator may have decreased trust and willingness to share the full range of perspectives on the topics covered.

Despite these limitations, and both deep and surface changes to selected materials, we postulate that the intervention should still be effective given the core elements (i.e., materials, coaching call) remain essentially the same as does the underlying theoretical approach [3, 5–7]. Furthermore, altering the messages to be culturally tailored to AI/AN communities should make this adaptation more relevant to AI/AN participants [8–11]. However, we do not know how important the humor was to the original intervention’s effectiveness and that has been removed. Thus, a next step is to evaluate the adapted intervention among these populations to assess whether it remains effective. Additional steps regarding the adaptation of the Smoke Free Homes intervention could include conducting focus group with urban AI/AN communities to assess relevance of the current adaptation to their context, and assessing the applicability of the content changes for indigenous communities outside of the U.S.

## Conclusion

Our adaptation study has potential to inform both research and practice about how to undertake cultural adaptations when scaling up an EBI for different populations. The remaining steps in the adaption process are to pilot test, implement and evaluate the adapted intervention [25, 36]. Ideally, this would include an evaluation of the impact of the adapted intervention in comparison with the original Smoke Free Homes: Some Things Are Better Outside intervention. Plans for these steps are currently underway. It is our hope that this description of the process will be useful for others considering the adaptation of EBI for use in AI/AN communities, or for use with other indigenous cultures that utilize sacred or traditional tobacco. Interventions such as this one have the potential to protect children and nonsmokers from SHS in single-family dwellings. Given tribal housing is excluded from the U.S. Department of Housing and Urban Development’s smoke-free rule for conventional public housing [37], this type of intervention can also be beneficial for families living in multi-unit housing. Cultural adaptations such as the one described here have the potential to strengthen current efforts to reduce the tobacco burden.

## Additional file

Additional file 1 Standards for Reporting Qualitative Research Checklist. (DOCX 21 kb)

## Data Availability

The datasets used and/or analyzed during the current study are available only with permission from all appropriate tribal governments.

## Abbreviations

AI/AN: American Indian/Alaska Native
EBI: Evidence-based intervention
NS: Nonsmoker
SFH: Smoke-free home
SHS: Secondhand smoke
